# The ethos brief index—validation of a brief questionnaire to evaluate wellness based on a holistic perspective in patients with restless legs syndrome

**DOI:** 10.1007/s11325-024-03058-5

**Published:** 2024-05-14

**Authors:** Susanne Knutsson, Maria Björk, Elzana Odzakovic, Amanda Hellström, Christina Sandlund, Martin Ulander, Jonas Lind, Bengt Fridlund, Amir Pakpour, Anders Broström

**Affiliations:** 1 Department of Health and Caring Sciences, Faculty of Health and Life Sciences, Linnaeus University, 351 95 Växjö, Sweden; 2 Center of Interprofessional Collaboration Within Emergency Care (CICE), Linnaeus University, Växjö, Sweden; 3 Department of Nursing, School of Health and Welfare, Jönköping University, Jönköping, Sweden; 4 Department of Health and Caring Sciences, Faculty of Health and Life Sciences, Linnaeus University, Kalmar, Sweden; 5 Department of Neurobiology, Care Sciences and Society, Karolinska Institutet, Huddinge, Sweden; 6 Academic Primary Health Care Center, Stockholm, Region Stockholm Sweden; 7 Department of Biomedical and Clinical Sciences, Division of Neurobiology, Linköping University, Linköping, Sweden; 8 Department of Clinical Neurophysiology, Linköping University Hospital, Linköping, Sweden; 9 Section of Neurology, Department of Internal Medicine, County Hospital Ryhov, Jönköping, Sweden; 10 Department of Health and Caring Sciences, Western Norway University of Applied Sciences, Bergen, Vestlandet Norway

**Keywords:** Restless legs syndrome, Sleep, Validity, Reliability, Ethos

## Abstract

**Purpose:**

The aim of this study was to validate the Ethos Brief Index (EBI) in patients with Restless Legs Syndrome (RLS).

**Methods:**

A cross-sectional design, including 788 subjects with RLS (65% women, 70.8 years, SD 11.3) from the Swedish RLS Association, was used. A postal survey was sent out to collect data regarding socio demographics, comorbidities, and RLS-related treatment data. Questionnaires included were EBI, the Restless Legs Syndrome-6 Scale (RLS-6), Restless Legs Syndrome—Quality of Life questionnaire (RLSQoL), the Insomnia Severity Index (ISI), and the Epworth Sleepiness Scale (ESS). The validity and reliability of the EBI were investigated using Rasch and confirmatory factor analysis (CFA) models. Measurement invariance, unidimensionality, and differential item functioning (DIF) across age and gender groups, as well as insomnia, daytime sleepiness, RLS-related QoL and RLS severity were assessed.

**Results:**

The results supported the unidimensionality of the EBI in the CFA (i.e., explaining 61.5% of the variance) and the Rasch model. The reliability of the EBI was confirmed using composite reliability and Cronbach’s alpha. No DIF was identified for gender, age, insomnia, daytime sleepiness, RLS severity or RLS-related QoL.

**Conclusion:**

The EBI showed good validity and reliability and operated equivalently for male and female patients with RLS. Accordingly, healthcare professionals can use the EBI as a psychometrically sound tool to explore and identify patient-centered problems related to the whole life situation.

## Introduction

Restless legs syndrome (RLS)/Willis-Ekbom disease is a highly prevalent (i.e., 3% worldwide prevalence; [1] neurologic, sensory-motor circadian rhythm disorder [2] characterized by an involuntary desire to move the limbs. The patients’ degree of discomfort ranges from mild and infrequent to severe [3]. Pronounced long-term sleep problems, daytime sleepiness and fatigue are common, as well as cognitive deficits and depressive symptoms [4], which in many cases affect the whole life situation [5]. Five diagnostic criteria are used to set the diagnosis [6] (Table 1).

**Table 1:** The five diagnostic criteria for restless legs syndrome set by the International Restless Legs Syndrome Study Group (IRLSSG) [6]

Criteria	Description
I	Desire to move the limbs, which usually are associated with paresthesias/dysesthesias
II	Motor restlessness
III	Symptoms, worse or exclusively present at rest (i.e., lying, sitting) with partial and temporary relief by activity
IV	Symptoms worse in evening/night
V	The symptoms are not only described as primary to another medical/behavioral condition but may be secondary to other diseases or conditions

Although pathophysiology is not fully understood, dopamine agonists, L-dopa, Alpha-2-delta ligands, opioids, or iron are potential treatment alternatives [7, 8]. Non-pharmacological treatments such as exercise, yoga, acupuncture, infrared devices, vibration pads, and cold air chambers also exist but need to be further evaluated [9, 10]. Both arriving at the correct diagnosis and finding the right treatment might take time. Consequently, Fulda et al., [11] recently emphasized the need to improve the availability and quality of instruments to measure the patient perspective, both before and after treatment initiation. RLS-specific instruments exist, either to measure symptom severity [12], or Quality of life (QoL) [13, 14]. Generic QoL instruments, in some cases extensive and burdensome for the patients, have also been used. A recent systematic review and meta-analysis including 27 studies conducted between 2000 and 2022 revealed total QoL, as well as physical and mental health components of QoL in patients with RLS to be low [15].

An alternative to RLS-specific and generic QoL instruments could be a questionnaire that evaluates Ethos toward wellness. Ethos as a concept stands for a multidimensional state of being, based on a holistic perspective, referring to an individual´s beliefs and motives regarding ethos toward health with a focus on well-being and QoL [16]. The 67-item Ethos Toward Wellness Questionnaire (EtWeQ) [16, 17] is grounded in theory [18–20] and has shown promising validity in a healthy population [16, 17], but it has not yet been used in people with RLS. One problem is that the EtWeQ is extensive, but the last part of the EtWeQ, the Ethos Brief Index (EBI), has proven valid in patients with obstructive sleep apnea (OSA) [21]. The EBI could, if found valid and reliable also in an RLS population, be a comprehensive tool for clinicians and researchers to evaluate Ethos before and after treatment initiation and could work as a holistic tool to strengthen the patient’s perspective during shared decision-making [22, 23]. The aim of this study was therefore to validate the EBI in patients with RLS.

## Methods

### Design and sample

A cross-sectional design was used. The study sample was derived from the Swedish RLS Association containing about 1500 members. All 1500 members were invited to participate in a postal survey. Inclusion criteria were 18 years of age or older, having a diagnosed and treated RLS, being able to speak and understand Swedish, and grant written informed consent. A total of 788 members returned the questionnaire, rendering a response rate of 52.5%.

### Data collection

Written information describing the project was sent by post to all listed members in May 2022, with one reminder in August 2022. They also received a separate document informing them that the board of the association had approved the dispatch. Acceptance of participation was indicated by returning the completed questionnaire in a pre-stamped envelope. Also, to make participation anonymous for those who wished, there was a separate envelope to include the signed informed consent when returning the questionnaire. Data on gender, age, employment, economic situation, years since diagnosis, and treatment aspects (i.e., self-reported comorbidities and treatment) were collected through the questionnaire to describe sample characteristics and to be used as covariates.

## Measures

### The EBI

The original version of the EBI [16, 17] includes nine items focusing on how satisfied the individual is with his/her work, family, housing, social life, financial situation, leisure time, living habits, lifestyle, and health and one question about the whole life situation. The items are scored on a scale from 0 (very bad) to 10 (very good). In the version tested in patients with obstructive sleep apnea [21], as well as in the present study, item 9 worded "I am satisfied with my total life situation" was deleted to decrease the number of items. The other eight items are summarized, yielding a total score of 0–80.

### Restless legs syndrome-6 scale (RLS-6)

The RLS-6 comprises four items about the severity of RLS symptoms and two items on sleep satisfaction and sleepiness during the past week. All items are rated on a 0–10 scale with higher scores indicating more problems. Item 5 refers to RLS mimics and can be used to differentiate RLS from other disorders [12].

### Restless legs syndrome – quality of life (RLSQoL)

The RLSQoL consists of 18 items capturing various aspects of daily activity, morning and evening activity, concentration, sexual activity, and work over the previous four weeks. Ten of the items contribute to a summary score (items 1–5, 7–10 and 13), the overall life impact score. Lower scores indicate lower QoL [13].

### The insomnia severity index (ISI)

The well-validated ISI contains seven items capturing the main symptoms of insomnia and impairments of daytime functioning during the past two weeks. A total score (0–28) is calculated, where higher scores indicate more insomnia symptoms [24].

### Epworth sleepiness scale (ESS)

The ESS assesses the proneness to doze off or fall asleep in eight specific situations. The scoring of each item is 0–3, where higher scores indicate a higher likelihood of falling asleep. The scores for each of the eight situations are added together to give a total score between 0 and 24 [25].

## Statistical analyses

Descriptive statistics were used to show patient characteristics. Item distributions and the percentages of missing data for each item were used to determine the feasibility of the EBI. A feasible scale should have < 10% of missing data for each item, an average score close to the scale midpoint, and should span most of its potential range, with floor/ceiling effects not exceeding 50%. The internal consistency of the EBI was assessed using Cronbach’s alpha, McDonald’s omega, and item-total correlations (corrected for overlap), with values higher than 0.7 for Cronbach’s alpha and McDonald’s omega, and 0.3 for item-total correlations, indicating satisfactory reliability [26].

### Confirmatory factor analysis (CFA)

Since theory on the construct already exists [16, 17, 21], the structural validity of the EBI was assessed by CFA using the diagonally weighted least squares estimator. Several indices were used to test the unidimensional structure of the EBI: comparative fit index (CFI), Tucker-Lewis index (TLI), root mean square error of approximation (RMSEA), and standardized root mean square residual (SRMR). Model fit was considered acceptable if CFI and TLI values exceeded 0.90, while RMSEA and SRMR values were < 0.08.

### Rasch measurement theory (RMT)

The unidimensionality of the EBI was further explored by Rasch analysis using the Partial Credit Model. Item fit was assessed using Infit and outfit mean square (MnSq), with values between 0.5 and 1.54 indicating acceptable fit. Local dependency is relative to the parameters of the specific dataset, and several factors may affect the value. For this reason, local dependency should be considered relative to the average residual correlation. In the current study, response dependence between items was examined using the residual correlation matrix where residual correlations > 0.2 above the average correlation were considered to indicate local dependency [27]. The principal component analysis (PCA) of the residuals was conducted in the Rasch analysis to examine unidimensionallity [28].

### Differential item functioning (DIF)

DIF was conducted across gender, age, insomnia, daytime sleepiness, RLSQoL and RLS-6 (excluding item 5) subgroups to determine if each item of the EBI exhibited invariance across these groups. A DIF > 1.0 logit indicated a notable DIF [29].

### Network analyses

To evaluate the connection between the EBI items and overall life satisfaction, two independent network analyses were conducted, one for working participants and one for retirees. The network was estimated using a graphical least absolute shrinkage and selection operator (LASSO) method based on the Extended Bayesian Information Criterion (EBIC) [30]. The network stability was assessed by a nonparametric bootstrapping method with 1000 replicates.

An independent t-test was employed to analyze the distinctions in EBI scores (i.e., dichotomized into low vs. high total score, based on the median EBI total score) concerning age, total life satisfaction, ESS, ISI, RLSQoL, and RLS-6 across worker and retired subsamples. Pairwise deletion was employed for both CFA and Rasch models. No imputation was done. Descriptive statistics were performed using SPSS (version 27.0) and JASP (version 0.17.1). CFA was performed in MPLUS (version 7.0) and the Rasch analysis using Winsteps (version 4.3.0). The significance level was set at > 0.05 for all analyses.

## Results

### Study population

Patient demographics and clinical characteristics of the 788 participants (70.8 years, SD 11.4) are shown in Table 2. There was a slight majority of women in the sample (65%) and most participants were retired (71%) with a mean age of 70.8 years. The mean time since diagnosis was 19.2 years and most had a pharmacological treatment, where dopamine agonist was most common. RLS-related sleep problems such as low sleep satisfaction and feeling tired during the day were frequently reported (Table 2).

**Table 2:** Characteristics of the study participants (n = 788)

Variables	Value
Female, n (%)	510 (65)
Age, mean, (SD)	70.8 (11.3)
Educational level, n (%)	
9 years or below 12–13 years University	166 (21) 277 (35) 345 (44)
Retired, yes, n (%)	560 (71)
Living together, yes, n (%)	582 (75)
Other diagnoses, n (%)	
Renal disease Parkinson’s disease Multiple sclerosis Migraine Iron deficiency	15 (2) 5 (0.5) 9 (1) 59 (7) 78 (10)
Pharmacological treatment, n (%)	
Dopamine agonists Opioids α2δ Ligands Dopa/derivates Iron supplement	625 (79) 163 (21) 144 (18) 105 (13) 33 (4)
Years since diagnosis, mean (SD)	19.2 (75.6)
RLS-6, mean (SD)	
Sleep satisfaction Problems falling asleep Problems during the night Daytime problems during resting Daytime problems during activity Tiredness/sleepiness during the day	5.9 (2.5) 4.6 (3.9) 4.9 (2.8) 4.5 (3.3) 1.6 (1.9) 5.2 (2.7)

RLS-6 = Restless Legs Syndrome-6 Scale

### Item distribution

The distribution of responses to the EBI items is shown in Table 3. The highest levels of satisfaction were reported for family (item 2), housing (item 3), and social life (item 4), while satisfaction with work (item 1) and health (item 8) had the lowest levels. Additionally, there was a considerable number of missing responses for item 1, due to the notable percentage of participants who were retired (71.1%).

**Table 3:** Distribution of responses, n and (%), to separate items included in the Ethos Brief Index (EBI) (n = 788)

Item #	0	1	2	3	4	5	6	7	8	9	10	Missing
I am satisfied with my:												
1. Work	1 (0.1)	4 (0.5)	4 (0.5)	13 (1.6)	11 (1.4)	20 (2.5)	16 (2.0)	41 (5.2)	77 (9.8)	57 (7.2)	70 (8.9)	474 (60.2)
2. Family	0	4 (0.5)	5 (0.6)	9 (1.1)	9 (1.1)	30 (3.8)	25 (3.2)	53 (6.7)	104 (13.2)	128 (16.2)	367 (46.6)	54 (6.9)
3. Housing	1 (0.1)	5 (0.6)	2 (0.3)	9 (1.1)	7 (0.9)	17 (2.2)	11 (1.4)	46 (5.8)	97 (12.3)	162 (20.6)	414 (52.5)	17 (2.2)
4. Social life	0	8 (1.0)	5 (0.6)	13 (1.6)	20 (2.5)	42 (5.3)	41 (5.2)	73 (9.3)	141 (17.9)	132 (16.8)	297 (37.7)	16 (2.0)
5. Financial situation	1 (0.1)	11 (1.4)	8 (1.0)	21 (2.7)	24 (3.0)	36 (4.6)	49 (6.2)	65 (8.2)	140 (17.8)	153 (19.4)	266 (33.8)	14 (1.8)
6. Leisure time	1 (0.1)	7 (0.9)	12 (1.5)	31 (3.9)	25 (3.2)	68 (8.6)	50 (6.3)	89 (11.3)	134 (17.0)	137 (17.4)	215 (27.3)	19 (2.4)
7. Lifestyle/ habits	1 (0.1)	8 (1.0)	13 (1.6)	30 (3.8)	32 (4.1)	69 (8.8)	70 (8.9)	80 (10.2)	164 (20.8)	125 (15.9)	182 (23.1)	14 (1.8)
8. Health	1 (0.1)	44 (5.6)	51 (6.5)	82 (10.4)	79 (10.0)	119 (15.1)	97 (12.3)	95 (12.1)	115 (14.6)	54 (6.9)	38 (4.8)	13 (1.6)

0 is very dissatisfied and 10 is very satisfied

### Internal consistency

The internal consistency of the EBI was satisfactory as evidenced by high values for the Cronbach’s α (0.87) and omega coefficient (ω = 0.87) (Table 4). Furthermore, all corrected item-total correlations were above 0.40 (Table 5). The one-factor structure was confirmed by the CFA: χ^2^ = 66.72 (df = 20), CFI = 0.986, TLI = 0.980, RMSEA = 0.055 and SRMR = 0.060. All factor loadings were significant and > 0.5.

**Table 4:** Psychometric properties of the Ethos Brief Index (EBI) at the scale level (n = 760)

Psychometric testing	Value	Suggested cutoff
Internal consistency (Cronbach’s α)	0.871	> 0.7
Omega coefficient **ω**	0.872	> 0.7
Average variance extracted (AVE)	0.458	> 0.5
Confirmatory factor analysis		
χ^2^ (*df*)	66.718 (20)	Nonsignificant
Comparative fit index	0.986	> 0.9
Tucker-Lewis index	0.980	> 0.9
Root mean square error of approximation	0.055	< 0.08
Standardized root mean square residual	0.060	< 0.08
Item separation reliability from Rasch	1.0	> 0.7
Item separation index from Rasch	14.48	> 2
Person separation reliability from Rasch	0.75	> 0.7
Person separation index from Rasch	1.75	> 2

**Table 5:** Psychometric properties of Ethos Brief Index (EBI) at the item level (n = 760)

Item #	Item score, Mean (SD)	Classical test theory analyses		Rasch analyses								
I am satisfied with my		Factor loading^a^	Item-total correlation	Infit MnSq	Outfit MnSq	Difficulty	DIF contrasts across gender groups^cd^	DIF contrasts across age groups^ce^	DIF contrasts across insomnia groups^cf^	DIF contrasts across daytime sleepiness groups^cg^	DIF contrasts across RLS QoL groups^ch^	DIF contrasts across RLS severity groups^ci^
1. Work	7.66 (2.18)	0.626	0.597	1.14	1.19	0.19	-0.10	0.0	0.03	0	-0.05	-0.09
2. Family	8.72 (1.80)	0.537	0.594	1.51	1.50	-0.46	-0.02	0.31	0	-0.07	-0.13	-0.16
3. Housing	9.00 (1.64)	0.594	0.636	1.22	1.00	-0.67	-0.06	0.08	0	0	-0.10	-0.22
4. Social life	8.28 (2.00)	0.764	0.720	0.91	0.81	-0.19	-0.13	0.0	-0.09	-0.08	-0.06	-0.15
5. Financial situation	8.10 (2.16)	0.601	0.594	1.34	1.24	-0.10	0.0	-0.30	-0.09	0.04	-0.18	0
6. Leisure time	7.73 (2.24)	0.790	0.732	0.75	0.69	0.09	0.0	0.06	-0.04	0.05	0.04	0
7. Lifestyle/ habits	7.54 (2.33)	0.826	0.739	0.70	0.67	0.18	-0.02	0	0.05	0.0	0.03	0.08
8. Health	5.59 (2.45)	0.564	0.492	1.30	1.36	0.96	0.10	0	0.09	0	0.24	0.22

^a^Based on confirmatory factor analysis

^b^Using Pearson correlation

^c^DIF = differential item functioning; DIF contrast > 0.5 indicates substantial DIF

^d^DIF contrast across sexes = Difficulty for females—Difficulty for males

^e^DIF contrast across age groups = Difficulty for participants with older ages (i.e., ≥ 70.79)—Difficulty for participants with younger ages (i.e., < 70.79)

^f^DIF contrast across insomnia groups = Difficulty for participants with insomnia (i.e., ≥ 15)—Difficulty for participants without insomnia (i.e., < 15)

^g^DIF contrast across daytime sleepiness groups = Difficulty for participants with daytime sleepiness (i.e., ≥ 10)—Difficulty for participants without daytime sleepiness (i.e., < 10)

^h^DIF contrast across RLSQoL groups = Difficulty for participants with high quality of life (i.e., RLSQoL ≥ 23.04)—Difficulty for participants with low RLSQoL (i.e., RLSQoL < 23.04)

^i^DIF contrast across severity of the RLS groups = Difficulty for participants with high severity of the RLS (i.e., RLS-6 ≥ 5.4)—Difficulty for participants with low severity of the RLS (i.e., RLS-6 < 5.4)

MnSq = mean square error; DIF = differential item functioning

The main results of the Rasch analysis are shown in Table 5. All items of the EBI fitted well with the data. However, family (item 2) showed marginal fit. The most difficult item was the one measuring satisfaction with health (item 8, 0.96 logit) while housing was the easiest (item 3, -0.67 logit). The result of the PCA showed that the EBI explained 61.5% of the total variance, with only 8% unexplained variability for the first contrast. No local dependency was observed for the items of the EBI. No substantial DIF was detected across subgroups based on gender, age, insomnia, daytime sleepiness, RLS-related QoL and RLS symptoms.

### Concurrent validity

**Fig. 1:**
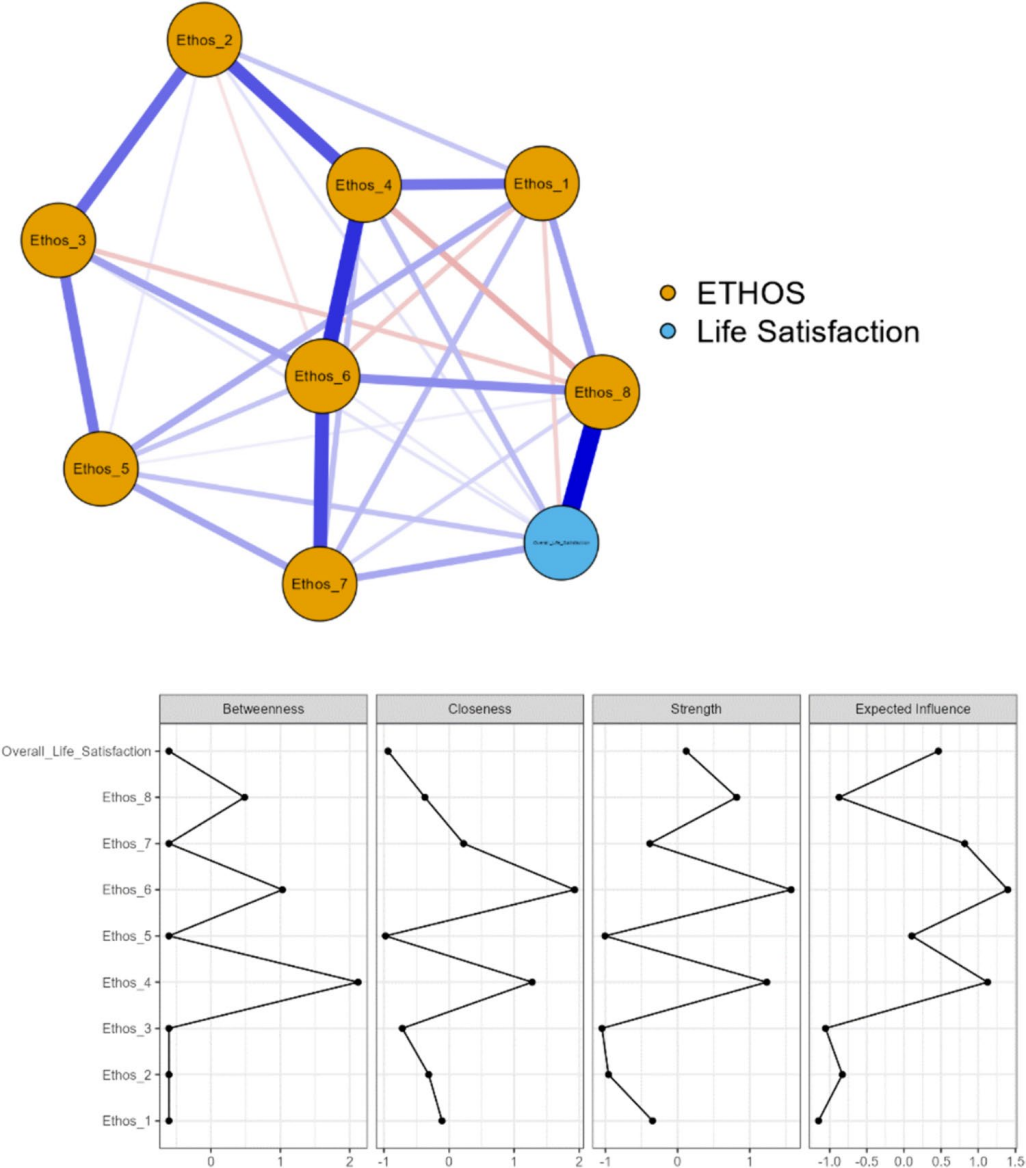
Network plot of the EBI items and overall life satisfaction in working participants (n = 200). Edges show partial correlations between the variables (nodes), where thicker edges represent stronger connections. Blue edges represent positive correlations, whereas red edges represent negative correlations. Note: Description of focus in the included EBI items: Item 1 = work, Item 2 = family, Item 3 = housing, Item 4 = social life, Item 5 = financial situation, Item 6 = leisure time, Item 7 = lifestyle/habits, Item 8 = health

**Fig. 2:**
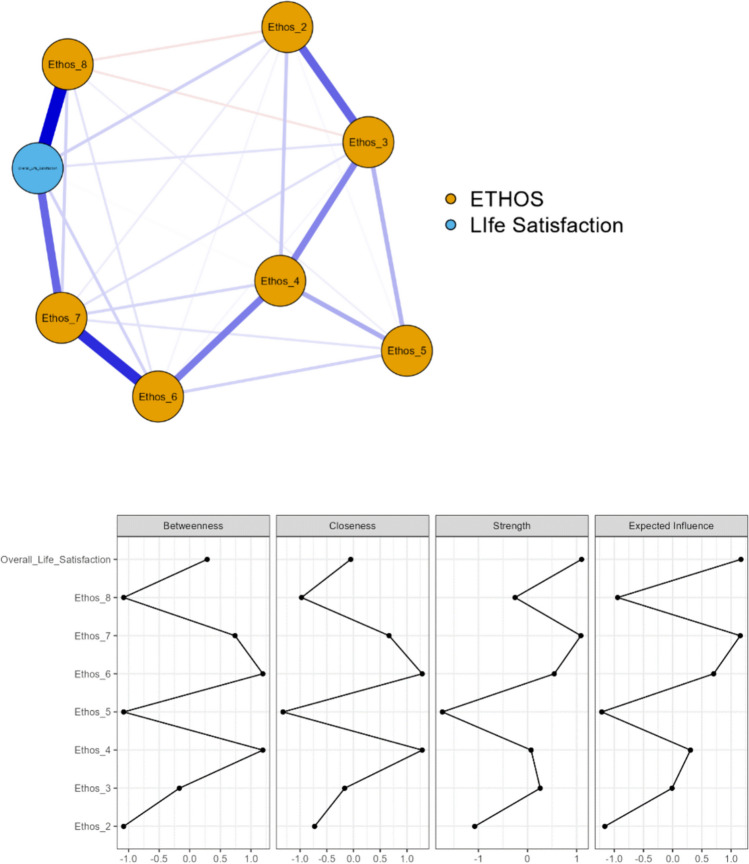
Network plot of the EBI items and overall life satisfaction in retired participants (n = 560). Edges show partial correlations between the variables (nodes), where thicker edges represent stronger connections. Blue edges represent positive correlations, whereas red edges represent negative correlations. Note: Description of focus in the included EBI items: Item 2 = family, Item 3 = housing, Item 4 = social life, Item 5 = financial situation, Item 6 = leisure time, Item 7 = lifestyle/habits, Item 8 = health

The relationships between the items in the EBI and overall life satisfaction were assessed by two network analyses (Figs. 1 and 2). In the network model for those who were working (Fig. 1), out of a possible 36 connections between the nodes, 31 connections were established, with a sparsity value of 0.139. Item 4 (social life) of the EBI showed the highest values across all centrality measures, the highest central variable, indicating the most central variable in network model 1. Conversely, items 1 (work), 2 (family) and 3 (housing) of the EBI showed low centrality measures. The strongest connections were observed between item 8 (health) of the EBI and overall life satisfaction (r = 0.474), item 4 (social life) and item 6 (leisure time) of the EBI (r = 0.387), and item 2 (family) and item 4 (social life) of the EBI (r = 0.315).

The network model for those who were retired (Fig. 2) showed 26 out of 28 possible edges/links and a sparsity value of 0.071. In this network model, item 4 (social life) and item 6 (leisure time) of the EBI showed the highest values across centrality measures, while items 2 (family), 5 (financial situation) and 8 (health) demonstrated the lowest centrality measures. The strongest connections were observed between item 8 (health)of the EBI and overall life satisfaction (r = 0.537), item 6 (leisure time) and item 7 (lifestyle/ habits) of the EBI (r = 0.445), item 2 (family) and item 3 (housing) of the EBI (r = 0.327), and item 7 (lifestyle/habits) of the EBI and overall life satisfaction (r = 0.325).

As presented in Table 6, workers with high EBI scores reported significantly higher overall life satisfaction and RLSQoL compared to those with low EBI scores. Retirees with high EBI scores reported significantly higher overall life satisfaction and RLSQoL, along with lower insomnia, sleepiness, and severity of RLS symptoms, in comparison to those with low scores. Interestingly, while age differences were observed among workers, those scoring high on the EBI being younger than those with low EBI scores, no significant age differences were observed among retirees.

**Table 6:** Comparisons of high and low EBI scores between workers and retirees across measures of total life satisfaction, RLS-related Quality of Life, RLS severity, daytime sleepiness, insomnia, and age (n = 760)

	Workers’ subsample ^a^ (n = 200)		t(p value)	Retired subsample ^b^ (n = 560)		t(p value)
	Low (n = 102) Mean ± SD	High (n = 93) Mean ± SD		Low (n = 281) Mean ± SD	High (n = 273) Mean ± SD	
Individual’s total life satisfaction	5.85 ± 2.08	8.19 ± 1.82	-8.31 (p < 0.001)	5.74 ± 2.14	8.34 ± 1.49	-16.80 (< 0.001)
Insomnia Severity Index	21.20 ± 5.24	19.93 ± 5.29	1.66 (0.098)	22.24 ± 4.83	18.96 ± 5.95	6.95 (< 0.001)
Restless Legs Syndrome – Quality of Life	59.027 ± 21.37	67.32 ± 19.86	-2.80 (0.006)	55.22 ± 22.45	71.01 ± 19.64	-8.77 (< 0.001)
Restless Legs Syndrome-6 Scale	5.05 ± 174	4.91 ± 1.91	0.50 (0.62)	5.59 ± 2.13	4.44 ± 2.02	6.41 (< 0.001)
Epworth Sleepiness Scale	10.34 ± 5.22	9.13 ± 5.57	1.54(0.13)	11.01 ± 5.80	8.71 ± 4.84	4.86 (< 0.001)
Age	60.84 ± 12.93	56.75 ± 9.87	2.49(0.013)	75.00 ± 8.17	75.54 ± 6.62	-0.88 (0.38)

^a^The EBI total score was dichotomized based on the median score, with scores of 63 or below categorized as 'low' Ethos and scores above 63 categorized as 'high' Ethos

^b^The EBI total score was dichotomized based on the median score, with scores of 56 or below categorized as 'low' Ethos and scores above 56 categorized as 'high' Ethos

## Discussion

This study, the first to validate an instrument used to measure Ethos in patients with RLS, found that the EBI showed good validity and reliability and operated equivalently for male and female patients with RLS. Specifically, unidimensionality of the EBI was identified in both the CFA and the Rasch model, while reliability was confirmed using composite reliability and Cronbach’s alpha. In addition, no DIF was identified for either sex, age, or any of the RLS-related variables (i.e., insomnia, daytime sleepiness, RLS severity, or RLS-related QoL), which makes EBI a short and valid alternative to RLS-specific and generic QoL instruments.

The unidimensional structure of the EBI found in both general [16, 17] and OSA populations [21] was also supported in the present study by results from both the CFA (i.e., explaining 61.5% of the variance) and Rasch analysis. Importantly, the internal consistency was satisfactory, as evidenced by high values of Cronbach’s α and omega coefficient. Out of the eight items, the one measuring satisfaction with health was the most difficult one, while satisfaction with housing was the easiest. No DIF was identified for gender, age, insomnia, daytime sleepiness, RLS severity or RLS-related QoL. When conducting analyses of separate groups (i.e., working vs. retirees), network analyses revealed a strong association of overall life satisfaction and satisfaction with health in both groups. Furthermore, comparisons of patients with high and low EBI scores in workers and retirees showed significant differences between the groups, with those with high Ethos having less severe RLS symptoms, less insomnia and daytime sleepiness, as well as better RLS-related QoL.

When investigating concurrent validity, network analysis showed a strong relationship between overall life satisfaction and satisfaction with health among those who were working. Moreover, strong relationships were also found between satisfaction with family, social life, leisure time and lifestyle. However, work, housing and financial situation had considerably less importance in relation to overall life satisfaction. Among those who were retired, the network analysis revealed the same strong relationship between overall life satisfaction and satisfaction with health. A strong relationship was also observed between satisfaction with family, housing, social life, leisure time and lifestyle. However, satisfaction with lifestyle and leisure time had in this case the strongest relationship with overall life satisfaction. One explanation might be that when retired, one may have more time for and satisfaction with leisure activities, and lifestyle becomes more important. Notably, as shown in both the present network analyses, and as described in another study [5], a long-term condition like RLS, seems, as anticipated to have a significant impact on satisfaction with health [19], which might lead to family and friends becoming increasingly important and material things and work less important [18]. Therefore, RLS-related symptoms may require a noticeable change of lifestyle and adaptation of activities [9] which may be important to be able to carry out activities. Support can therefore be of great importance for opportunities to carry out leisure activities. Certain leisure activities might be more associated with happiness. When mentally engaged in a pleasurable leisure activity, and distracted, a person with RLS might get symptom relief [9, 10] and in the long run achieve better satisfaction with various aspects of life. Retirees might also have had RLS for a long time and may have learned how to live with their disease and develop habits around intake of medication, use of self-care activities, and when to exercise. Furthermore, our comparisons between groups with high vs. low EBI scores showed that the retirees with a high score experienced better sleep. This may be due to being retired and maybe having fewer time constraints and more time to catch up on poor sleep.

The results showed that people who displayed a low EBI score also displayed more severe symptoms related to RLS. Furthermore, patients who displayed a high score consequently displayed less severe symptoms related to RLS. The EBI is short and easy to use, but an even simpler alternative could be to use item 9, which focuses on overall life satisfaction. If a person with RLS indicates low life satisfaction on item 9, there is, based on our findings, a possibility of finding low satisfaction in several of the other eight EBI items, as well as severe RLS symptoms. However, the full eight-item version is preferable as it gives a more nuanced picture of ethos. Consequently, the EBI could, supported by other tools measuring symptoms related to RLS itself (e.g., [12]), or sleep-related consequences (e.g., [24, 25]), be used as a patient-centered tool before and after initiation of RLS treatment. A shared decision-making approach [22], including the use of EBI as a holistic patient-centered assessment tool, might facilitate patient involvement in clinical care situations [23] both when a RLS diagnosis is set and when treatment is initiated and evaluated.

Several study limitations must be considered. Firstly, the sample (n = 788) contained mostly women with a high proportion of the total sample being retired. The relatively large dropout rate, as well as the effects of the above-mentioned socio-demographic factors, might have influenced the response pattern in the EBI, as well as in some of the other instruments. Secondly, data were collected through the nationwide Swedish RLS Association using a cross-sectional design. This limited the possibility of performing test–retest analyses, of exploring EBI change over time in relation to various treatment interventions, but also of providing results generalizable to a clinical setting. Future studies could address this by using a longitudinal design with clinical primary care and neurological outpatient data, collected before and after treatment has been initiated to evaluate EBI change over time in relation to various RLS-related symptoms and alternative interventions. Thirdly, all treatment-related variables regarding RLS, including assessments used for DIF, were self-reported. Therefore, recall bias is a problem regarding pharmacological treatment, comorbidities, the severity level of RLS, but also the severity level of other symptoms, such as insomnia and daytime sleepiness.

## Conclusion

The present study shows that all eight items of the EBI were embedded in one-factor measuring Ethos. The index showed good validity and reliability and operated equivalently across male and female patients with RLS. Accordingly, healthcare professionals can use the EBI as a psychometrically brief tool to explore and identify patient-centered problems related to the whole life situation, both before and after treatment initiation.

## Data Availability

Data will be made available on reasonable request.

## References

[1] Broström A, Alimoradi Z, Lind J, Ulander M, Lundin F, Pakpour A (2023) Worldwide estimation of restless legs syndrome: a systematic review and meta-analysis of prevalence in the general adult population. J Sleep Res 32:e13783. 10.1111/jsr.13783 36600470 10.1111/jsr.13783

[2] Lv Q, Wang X, Asakawa T, Wang XP (2021) Pharmacologic Treatment of Restless Legs Syndrome. Curr Neuropharmacol 19:372–382. 10.2174/1570159X19666201230150127 33380302 10.2174/1570159X19666201230150127 PMC8033969

[3] Khachatryan SG, Ferri R, Fulda S, Garcia-Borreguero D, Manconi M, Muntean M-L et al (2022) Restless legs syndrome: Over 50 years of European contribution. J Sleep Res 31:e13632. 10.1111/jsr.13632 35808955 10.1111/jsr.13632 PMC9542244

[4] Lee HB, Hening WA, Allen RP, Kalaydjian AE, Earley CJ, Eaton WW et al (2008) Restless legs syndrome is associated with DSM-IV major depressive disorder and panic disorder in the community. J Neuropsychiatry Clin Neurosci 20:101–105. 10.1176/jnp.2008.20.1.101 18305292 10.1176/jnp.2008.20.1.101

[5] Harrison EG, Keating JL, Morgan PE (2021) The experience of living with restless legs syndrome: A qualitative study. J Health Psych 26:1154–1167. 10.1177/1359105319871632 10.1177/1359105319871632 31434518

[6] Allen RP, Picchietti DL, Garcia-Borreguero D, Ondo WG, Walters AS, Winkelman JW, Zucconi M, Ferri R, Trenkwalder C, Lee HB, International Restless Legs Syndrome Study Group (2014) Restless legs syndrome/Willis-Ekbom disease diagnostic criteria: updated International Restless Legs Syndrome Study Group (IRLSSG) consensus criteria, history, rationale, description, and significance. Sleep Med 15:860–73. 10.1016/j.sleep.2014.03.025 25023924 10.1016/j.sleep.2014.03.025

[7] Garcia-Borreguero D, Cano-Pumarega I, Marulanda R (2018) Management of treatment failure in restless legs syndrome (Willis-Ekbom disease). Sleep Med Rev 41:50–60. 10.1016/j.smrv.2018.01.001 29602660 10.1016/j.smrv.2018.01.001

[8] Winkelman JW (2022) Treating severe refractory and augmented restless legs syndrome. Chest 162:693–700. 10.1016/j.chest.2022.05.014 35609673 10.1016/j.chest.2022.05.014

[9] Harrison EG, Keating JL, Morgan PE (2019) Non-pharmacological interventions for restless legs syndrome: a systematic review of randomised controlled trials. Disabil Reh 41:2006–2014. 10.1080/09638288.2018.1453875 10.1080/09638288.2018.1453875 29561180

[10] Guay A, Houle M, O’Shaughnessy J, Descarreaux M (2020) Current evidence on diagnostic criteria, relevant outcome measures, and efficacy of nonpharmacologic therapy in the management of restless legs syndrome (RLS): a scoping review. J Manipulative Physiol Ther 43:930–941. 10.1016/j.jmpt.2020.05.004 32900545 10.1016/j.jmpt.2020.05.004

[11] Fulda S, Allen RP, Earley CJ, Högl B, Garcia-Borreguero D, Inoue Y, Ondo W, Walters AS, Williams AM, Winkelman JW (2021) We need to do better: a systematic review and meta-analysis of diagnostic test accuracy of restless legs syndrome screening instruments. Sleep Med Rev 58:101461. 10.1016/j.smrv.2021.101461 33838561 10.1016/j.smrv.2021.101461

[12] Kohnen R, Martinez-Martin P, Benes H, Trenkwalder C, Hogl B, Dunkl E et al (2016) Rating of daytime and nighttime symptoms in RLS: validation of the RLS-6 scale of restless legs syndrome/Willis-Ekbom disease. Sleep Med 20:116–122. 10.1016/j.sleep.2015.10.014 27318235 10.1016/j.sleep.2015.10.014

[13] Abetz L, Vallow SM, Kirsch J, Allen RP, Washburn T, Earley CJ (2005) Validation of the restless legs syndrome quality of life questionnaire. Value Health 8:157–167. 10.1111/j.1524-4733.2005.03010.x 15804324 10.1111/j.1524-4733.2005.03010.x

[14] Abetz L, Allen R, Follet A, Washburn T, Earley C, Kirsch J, Knight H (2004) Evaluating the quality of life of patients with restless legs syndrome. Clin Ther 26:925–935. 10.1016/s0149-2918(04)90136-1 15262463 10.1016/s0149-2918(04)90136-1

[15] Broström A, Alimoradi Z, Odzakovic E, Kaldo V, Jernelöv S, Lind J, Ulander M, Pakpour A (2024) Quality of life among patients with restless legs syndrome: A systematic review and meta-analysis. J Clin Neurophys 122:80–91. 10.1016/j.jocn.2024.02.027 10.1016/j.jocn.2024.02.027 38489955

[16] Fridlund B, Baigi A (2014) Developing and establishing the psychometric properties of an Ethos Towards Wellness Questionnaire (EtWeQ). Open J Nurs 5:1–10. https://www.scirp.org/pdf/OJN_2014062014091410.pdf. Accessed 10 May 2024 10.1177/0898010115572632 25749994

[17] Fridlund B, Mårtensson J, Baigi A, Broström A (2015) Establishing the psychometric properties of the comprehensive Ethos Towards Wellness Questionnaire in a Norwegian population. J Holist Nurs 33:366–373. 10.1177/0898010115572632 25749994 10.1177/0898010115572632

[18] Corbin CB, Pangrazi RP (2005) Toward a uniform definition of wellness. Research Digest 3:1–8. https://www.researchgate.net/publication/234720652_Toward_a_Uniform_Definition_of_Wellness_A_Commentary. Accessed 10 May 2024

[19] Saylor C (2004) The circle of health: a health definition model. J Hol Nurs 22:97–115. 10.1177/0898010104264775 10.1177/0898010104264775 15154987

[20] Miller G, Foster LT (2010) Critical synthesis of wellness literature. Faculty of human and social development, University of Victoria, Victoria. https://dspace.library.uvic.ca/server/api/core/bitstreams/6b8c01d8-b639-439a-af1d-d448cf943263/content. Accessed 10 May 2024

[21] Broström A, Pakpour AH, Nilsen P, Fridlund B, Ulander M (2019) Psychometric properties of the Ethos Brief Index (EBI) using factorial structure and Rasch Analysis among patients with obstructive sleep apnea before and after CPAP treatment is initiated. Sleep Breath 23:761–768. 10.1007/s11325-018-1762-z 30523558 10.1007/s11325-018-1762-z PMC6700038

[22] Elwyn G, Frosch D, Thomson R, Joseph-Williams N, Lloyd A, Kinnersley P, Cording E, Tomson D, Dodd C, Rollnick S, Edwards A, Barry M (2012) Shared decision making: a model for clinical practice. J Gen Intern Med 27:1361–1367. 10.1007/s11606-012-2077-6 22618581 10.1007/s11606-012-2077-6 PMC3445676

[23] Björk M, Knutsson S, Odzakovic E, Hellström A, Sandlund C, Ulander M, Lind J, Pakpour AH, Broström A, members of the Jönköping University (JU) Sleep Well Research Group (2023) Validation of two brief instruments (the SURE and CollaboRATE) to measure shared decision-making in patients with restless legs syndrome. J Sleep Res 1:e14071. 10.1111/jsr.14071. (Online ahead of print. PMID: 37909257) 10.1111/jsr.14071 37909257

[24] Bastien CH, Vallières A, Morin CM (2001) Validation of the insomnia severity index as an outcome measure for insomnia research. Sleep Med 2:297–307. 10.1016/S1389-9457(00)00065-4 11438246 10.1016/S1389-9457(00)00065-4

[25] Johns MW (1991) A new method for measuring daytime sleepiness: the Epworth sleepiness scale. Sleep 14:540–545. 10.1093/sleep/14.6.540 1798888 10.1093/sleep/14.6.540

[26] Ferketich S (1991) Focus on psychometrics. Aspects of item analysis. Res Nurs Health 14:165–168. 10.1002/nur.4770140211 2047538 10.1002/nur.4770140211

[27] Christensen KB, Makransky G, Horton M (2017) Critical values for yen’s Q3: identification of local dependence in the rasch model using residual correlations. App Psych Meas 41:178–194. 10.1177/0146621616677520 10.1177/0146621616677520 PMC5978551 29881087

[28] McCreary LL, Conrad KM, Conrad KJ, Scott CK, Funk RR, Dennis ML (2013) Using the Rasch measurement model in psychometric analysis of the family effectiveness measure. Nurs Res 62:149–159. 10.1097/NNR.0b013e31828eafe6 23636342 10.1097/NNR.0b013e31828eafe6 PMC3678382

[29] Lundstrom M, Pesudovs K (2009) Catquest-9SF patient outcomes questionnaire: Nine-item short-form Rasch-scaled revision of the Catquest questionnaire. J Cataract Refract Surg 35:504–513. 10.1016/j.jcrs.2008.11.038 19251145 10.1016/j.jcrs.2008.11.038

[30] Chen J, Chen Z (2008) Extended Bayesian information criteria for model selection with large model spaces. Biometrika 95:759–71. 10.1093/biomet/asn034, https://www.stat.ubc.ca/~jhchen/paper/Bio08.pdf. Accessed 10 May 2024

